# Molecularly Imprinted Polymeric Particles Created Using Droplet-Based Microfluidics: Preparation and Applications

**DOI:** 10.3390/mi14040763

**Published:** 2023-03-29

**Authors:** Sinem Orbay, Amitav Sanyal

**Affiliations:** 1 Institute of Biomedical Engineering, Bogazici University, Istanbul 34684, Turkey; sinem.orbay@boun.edu.tr; 2 Biomedical Engineering Department, Erzincan Binali Yildirim University, Erzincan 24002, Turkey; 3 Department of Chemistry, Center for Life Sciences and Technologies, Bogazici University, Istanbul 34342, Turkey

**Keywords:** molecularly imprinted polymers, droplet-based microfluidics, biomedical/chemical applications

## Abstract

Recent years have witnessed increased attention to the use of droplet-based microfluidics as a tool for the fabrication of microparticles due to this method’s ability to exploit fluid mechanics to create materials with a narrow range of sizes. In addition, this approach offers a controllable way to configure the composition of the resulting micro/nanomaterials. To date, molecularly imprinted polymers (MIPs) in particle form have been prepared using various polymerization methods for several applications in biology and chemistry. However, the traditional approach, that is, the production of microparticles through grinding and sieving, generally leads to poor control over particle size and distribution. Droplet-based microfluidics offers an attractive alternative for the fabrication of molecularly imprinted microparticles. This mini-review aims to present recent examples highlighting the application of droplet-based microfluidics to fabricate molecularly imprinted polymeric particles for applications in the chemical and biomedical sciences.

## 1. Molecularly Imprinted Polymers (MIPs)

**Figure 1:**
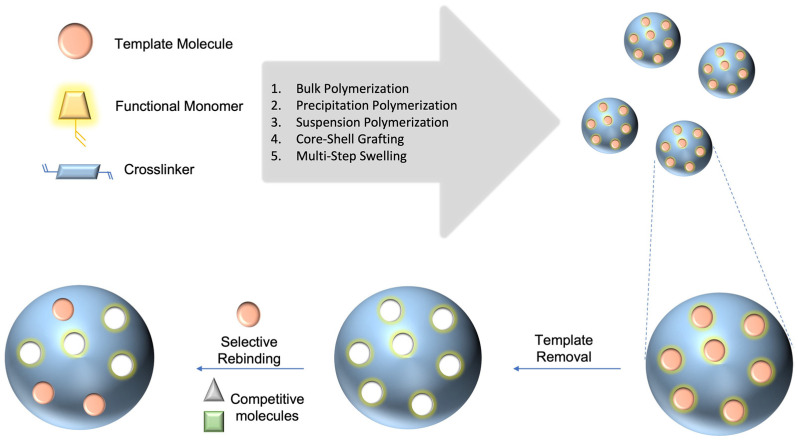
General demonstration and traditional preparation methods of MIPs.

The selective recognition of target molecules in a pool containing a vast quantity of similar molecules is required for several biological and chemical applications. Studies of the underlying mechanism of molecular recognition began in the 1800s when the “lock and key” theory, which describes the relationship between an enzyme and a substrate, was proposed [1]. A similar “fit-together” phenomenon is observed in the binding of a drug to a biological target [2], antigen–antibody interactions in the immune system [3], and the transcription process concerning the construction of mRNA from DNA [4]. In nature, the recognition of a target molecule occurs through a combination of non-covalent interactions, such as hydrogen bonding and weak electrostatic and hydrophobic interactions. Natural receptors demonstrate high selectivity and specificity to their respective counterparts; therefore, they are widely used for applications in analytical chemistry, diagnostics, and environmental studies. However, natural receptors are very expensive, entail complicated production steps, have limited lifetimes due to their facile degradation upon exposure to oxygen or microorganisms, and are often unsuitable for use with acids, bases, or organic solvents. Such drawbacks limit their widespread usage in many applications and have been the motivation for researchers to explore alternative synthetic approaches. Early studies by Mosbach demonstrated that the use of a target molecule as a template allowed for the creation of imprinted cavities in crosslinked polymers through the connection of different building blocks [5]. In the imprinting process, various associations, such as covalent bonding, hydrogen bonding, van der Waals forces, and hydrophobic or ionic interactions, are involved in keeping the building blocks associated with the template molecule, and different polymerization methods are used for the fixation of binding groups [6,7,8]. Thus, complexes are formed between the template and functional monomers through self-assembly, where the later components are crosslinked using a polymerization process. The overall process yields highly crosslinked polymeric materials, which preserve a binding pocket after the removal of the template, as illustrated in Figure 1.

The resulting molecularly imprinted polymeric materials have been explored in different applications, including solid phase extraction [9], catalysis [10], separation [11], sensing [12], and drug delivery [13], since their preparation methods are relatively straightforward, robust, and reliable. In addition, the numerous commercially available or synthetically accessible monomers enable the fabrication of materials that bear the potential to replace the utilization of natural receptors in many of these applications. However, heterogeneity in terms of size that results from the fabrication process limits their performance. Apart from the traditional approach of obtaining microparticles through the mechanical grinding of bulk monoliths obtained using bulk polymerization, contemporary polymerization, as discussed below, provides attractive alternatives for obtaining molecularly imprinted micro/nanoparticles. One of the latest focuses in imprinting studies is the improvement of new morphologies at the molecular level using different preparation methods because the influence of morphology on the applications of MIPs should be highlighted [14].

## 2. General Preparation Methods for MIP Microparticles

MIPs can be prepared using different methods. Non-covalent imprinting is the primary type of imprinting based on creating interactions between the target molecule and the functional monomers in a pre-polymerization mixture [15]. The strategies include bulk polymerization, precipitation polymerization, suspension polymerization, multi-step swelling, and core–shell imprinting. Each method has unique characteristics and various advantages and disadvantages, as summarized in Table 1.

The first method developed to synthesize MIPs was bulk polymerization. This is the most traditional and convenient method in studies due to its simplicity and universality [35]. It only requires mixing all components, including functional monomers, crosslinkers, initiator, and solvents; purging the mixture with nitrogen; and polymerizing it. The resulting polymer monolith must be crushed, ground, and sieved mechanically (generally with a mortar and pestle) to obtain approximately 20–50 micron-sized polymeric particles. The obtained particles have a wide range of molecular weights and sizes, and their binding sites are not homogenous. The irregular shapes of the resultant particles are unfavorable for chromatographic and separatory applications.

Moreover, the mechanical grinding step is time consuming and laborsome. During this process, the binding sites of the particles are destructed, which lowers MIPs’ loading capacity concerning theoretical values, and a large amount of the product is wasted (approximately 50% of the ground polymer). Additionally, traditional bulk polymerization is unsuitable for larger target biomolecules [36]. In the last few years, researchers have started synthesizing spherical MIPs as they are advantageous in terms of production and performance in further applications.

**Figure 2:**
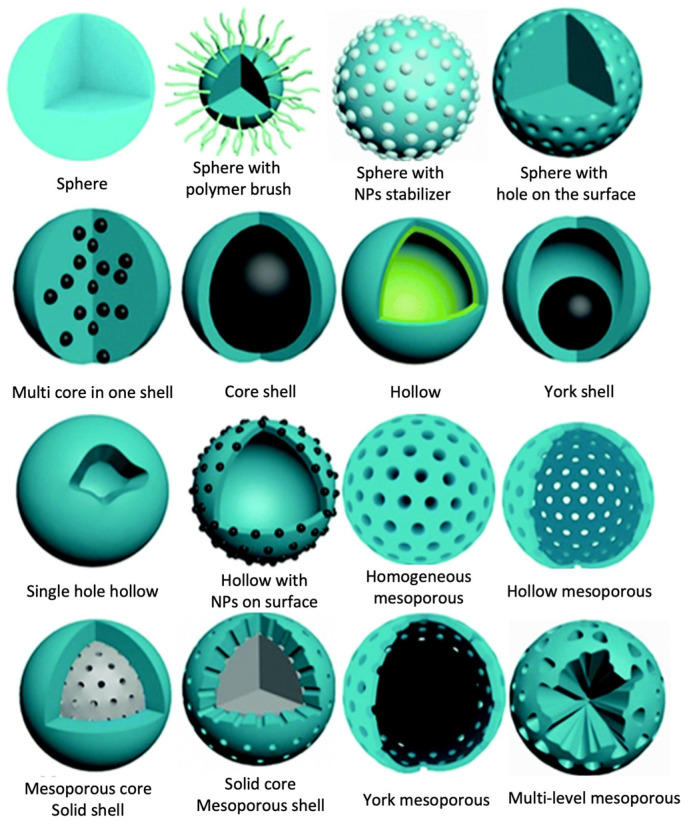
Different morphologies of spherical MIPs. Reproduced with permission from [14].

Over time, the morphology of MIPs has changed from irregular forms to spherical beads. As demonstrated in Figure 2, spherical MIP beads can be changed into numerous forms using different preparation methods [14]. In the literature, different MIP preparation methods for the development of controllable spherical morphologies have been introduced to create particles with higher-surface area-to-volume ratios and controllable sizes [37].

Precipitation polymerization is a straightforward, one-step method performed similarly to bulk polymerization except for the excessive amount of solvent used in the polymerization mixture [19,20,21,22]. This surfactant- and emulsifier-free method allows for the acquisition of uniform and spherical particles with diameters less than 1 mm under very dilute conditions. Particle formation transpires either when the polymer chains reach the solubility limit, and the resultant polymer particles precipitate from the solution, or when the crosslinking density is increased, thereby expelling the solvent from the polymeric chain, and causing the particles to collapse. The particles’ size and porosity can be tuned by adjusting the polymerization conditions. It has been proven that the resultant spherical particles formed through this process perform better than those obtained by bulk polymerization [16,38]. The drawback of this method is the need for large quantities of template molecules and solvent due to the dilution factor, but this can be compensated with higher yields.

Suspension polymerization is another common method for the direct preparation of MIPs in the form of spherical imprinted beads [23,24,25,26]. It is performed in a heterogeneous environment where water [39], mineral oil [40], or a perfluorocarbon liquid [41] are utilized to suspend the droplet of the polymerization mixture. In the suspension polymerization approach, it is necessary to use surfactants or emulsifiers to prevent the agglomeration and coalescence of monomer droplets. Theoretically, this method is an attractive alternative to the use of bulk polymers since the spherical morphology and higher yields of the resultant particles offer better chromatographic characteristics. In addition, it is possible to obtain relatively large particles within the range from micrometers to millimeters. However, in this two-phase system, using water or highly polar organic solvents as the continuous phase for hydrophobic monomers will lead to poor recognition since there will be competition between the solvent and functional monomers to build a specific interaction with the template molecule. This causes a decrease in the strength of the interaction between functional monomers and the print molecule. Therefore, the use of liquid perfluorocarbon as a dispersing phase was introduced in the literature as an approach that overcomes the competing solvent effect [42]. Multi-step swelling is another approach that is also hampered by the competing solvent effect. This method consists of an initial stepwise, preformed seed-particle-swelling procedure followed by polymerization [27,28,29]. During the swelling procedure, an aqueous phase is required, which interferes with template molecule–monomer interactions. Even though the obtained particles are relatively monodisperse in size and shape and, for this very reason, are well suited for chromatographic applications, the complex polymerization procedures and reaction conditions involved in their synthesis are not desirable.

Recently, molecular imprinting on the surface of nanomaterials with desired properties has emerged. This core–shell method can overcome the utilization rate problem concerning recognition sites in irregular and solid spherical MIPs. Core–shell-imprinted polymers integrated with other functional components, such as magnetic nanoparticles, quantum dots, gold nanoclusters, or silica, offer an attractive alternative approach [30,31,32,33,34]. MIPs can be utilized in core–shell construction as either the core or shell material. Despite the complex synthesis process, this technique results in products with higher adsorption capacity, faster mass transfer, and a uniform distribution of binding sites compared to the bulk method.

**Figure 3:**
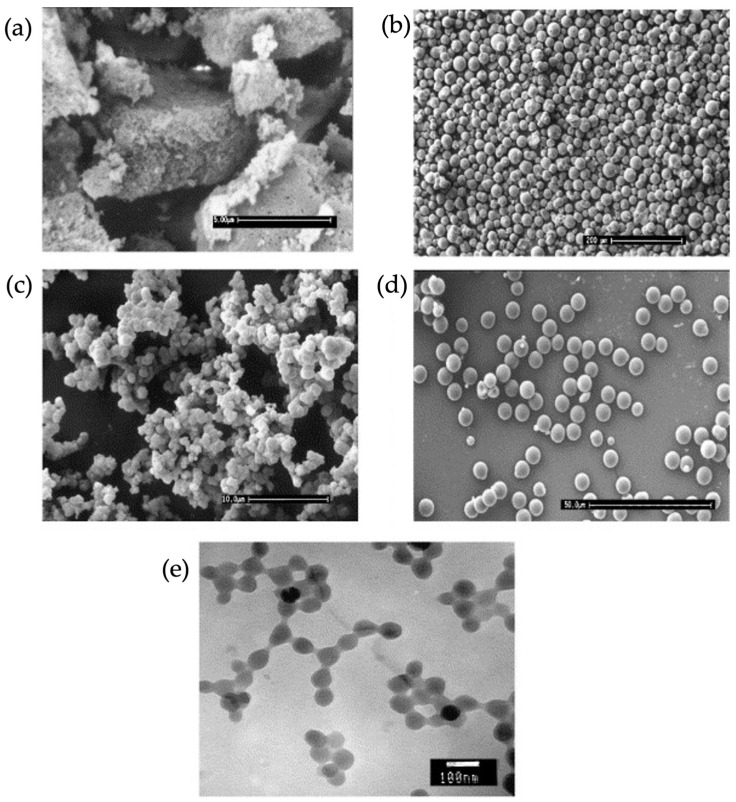
SEM and TEM images of the polymeric particles obtained through different polymerization methods. (**a**) Bulk Polymerization, (**b**) suspension Polymerization, (**c**) precipitation polymerization, (**d**) 2-step swelling, and (**e**) core–shell polymerization. Reproduced with permission from [47].

Different methodologies for the creation of imprinted polymers in the form of particles within the nano–micrometer size range have been introduced in the literature, and the resultant polymers are used in many applications [43,44]. Polymers created by different methods possess unique characteristics, and these methods offer divergent benefits. Comparison studies have reported how imprinted particles’ morphology and formation processes affect their final performance [45,46]. Moral et al. compared and tested different polymerization methods for imprinted polymer particles under the same conditions and investigated the rebinding performance of the resulting imprinted materials. The morphological differences of the resultant particles obtained from different polymerization approaches were characterized using SEM, as shown in Figure 3 [47].

Besides the conventional polymerization methods for polymeric particles’ synthesis, the use of droplet-based microfluidic apparatuses has started cropping up in the literature [48,49,50]. Such a device enables the control of the diameter and morphology of the beads according to the width and depth of the flow channels, flow rates, and the mixed ratio of continuous and dispersed phases. The resultant particles are highly uniform and monodisperse. In this review, we focus on the preparation of spherical MIP using droplet microfluidics, which constitutes a small fraction of the literature but offers great potential for integration with complex microfluidic setups and various advantages in the production process and in final applications.

## 3. Droplet-Based Microfluidics for the Preparation of MIPs and Their Applications

Droplet-based microfluidics deals with the generation, operation, and manipulation of droplets with micrometric dimensions [51,52]. The strength of droplet-based microfluidics is that enables the formation of uniform droplets and particles whose size, shape, and dispersity can be controlled. Additionally, its use allows one to decrease costs and minimize the required number of reagents and materials. Droplet formation occurs when two incompatible liquids flow into their respective microchannels at specific flow rates. When the two streams, as continuous and dispersed phases, meet at the intersection of the channels, the dispersed phase breaks up into a stream of droplets due to the shear force caused by the continuous phase. To date, microfluidic droplet generators have been fabricated using various substrates, including silicon, quartz, glass, ceramics, and polymers, wherein the desired application of the device governs the choice of substrate. In recent years, the soft-lithography-based microfabrication of these devices has become widespread, and today, an ever-increasing number of researchers have access to the facilities required to fabricate such devices or are able to purchase them from several commercial sources. The commercially available chips are generally produced using thermoplastic polymeric materials such as polycarbonate (PC), polystyrene (PS), cyclic olefin copolymer (COC), or polymethylmethacrylate (PMMA). Thermoplastics are preferred since they can be easily integrated with different components such as valves, reservoirs, and filters. However, they have limited stability against organic solvents, and not all thermoplastic polymers are suitable for use with mineral oil systems for droplet generation applications. PDMS microchannels, on the other hand, are compatible with many organic solvents, and PDMS-based droplet generation systems allow one to work with a large variety of oil phase components and surfactant combinations. 

**Figure 4:**
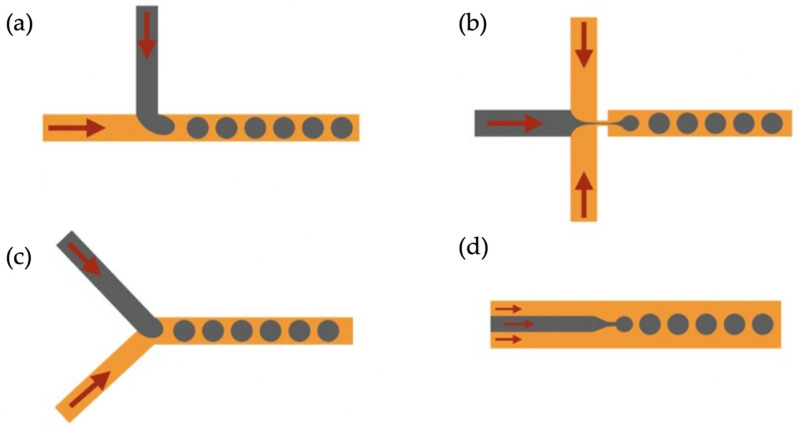
Different channel geometries for droplet generation. (**a**) T-junction, (**b**) alternating T-junction, (**c**) Y-junction, and (**d**) planar co-flow. Reproduced with permission from [53].

Although several possibilities of channel configurations are possible, traditionally, only four different channel geometries have been largely explored for droplet generation: T-junction, Y-junction, flow focusing, and co-flow geometry (Figure 4) [53].

Although the traditional methods for the fabrication of spherical MIP particles deliver reasonable results, they are not capable of generating monodisperse and size-controllable particles. In a droplet-based system, spherical and monodisperse particles are obtained by breaking the shearing force of the continuous phase. In T- and Y-junction designs, when the immiscible fluids meet at the intersection of the microfluidic channel, the dispersed phase progressively enters the main channel and forms droplets by breaking the continuous phase. On the other hand, in a co-flow system, the viscous stresses of the continuous phase break the dispersed phase as it enters the main channel. Through the flow rates, channel dimensions, and ratios of the continuous and dispersed phases, particle dimensions can be arranged and optimized. The selection of the channel design, formation mechanism (O/W or W/O), and curing method depends on the polymer system and the target molecule. 

**Figure 5:**
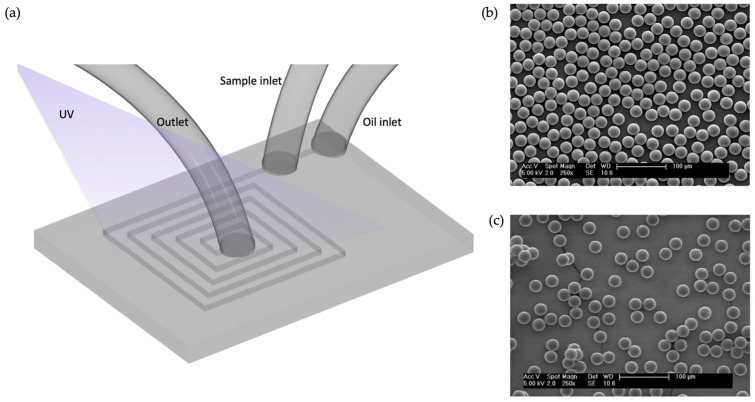
(**a**) General scheme of the spiral micro-reactor, (**b**) SEM images of beads produced in oil using the micro-reactor, and (**c**) SEM images of beads produced in liquid fluorocarbon using the micro-reactor. Reproduced with permission from [54].

The ability to create a monodisperse emulsion inside a droplet generator has been proposed to have applications in several research areas, including the preparation of MIPs. The methods described so far for the fabrication of spherical MIPs either use expensive reagents or employ complex polymerization methods. An alternative approach that uses a polycarbonate-based spiral microreactor was proposed by Zourob et al. (Figure 5a) [54]. This group designed a long, spiral channel with a length of almost 2 m and used a UV source for particle polymerization. The aim of the study was to demonstrate the broad applicability of MIP production methods. They proved this by producing highly monodispersed MIP microspheres, as seen in Figure 5b,c. They used the monodispersed particles to create a binding assay for ^3^H-propranol and used only 0.25 mg microparticles for their binding assay, which is quite promising for applications due to the low level of material consumption. Since the direct fabrication of spherical particles enables better performance in chromatography and solid-phase extraction applications than traditional methods, the authors intended to use the method for a further solid-phase extraction application. They used mineral oil without any surfactant as a continuous phase in the microreactor, constituting an approach that is less expensive than that used in a previously demonstrated study conducted by Mosbach and Mayes concerning a perfluorocarbon liquid [42]. This method can be considered a first step in the demonstration of the creation of MIPs inside a microfluidic device. Subsequently, depending on the experimental design and the final application, oil-in-water (O/W) or water-in-oil (W/O) droplets are produced by using microfluidics channels for spherical MIPs, as summarized in Table 2.

**Figure 6:**
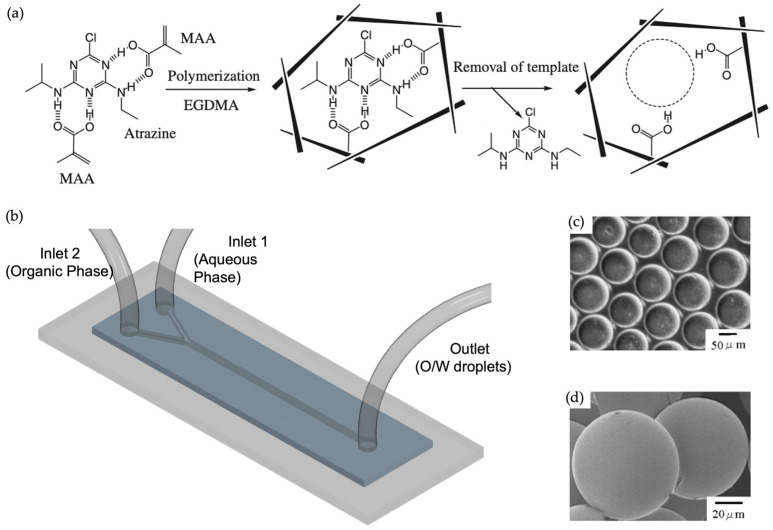
(**a**) Schematic demonstration of atrazine-imprinted polymers; (**b**) Y-junction microchannel with dimensions of 2 mm in length, 153.1 μm in width, and 80 μm in depth; (**c**) microscopic image of droplets before polymerization; (**d**) SEM image of atrazine-imprinted beads. Reproduced with permission from [55].

Takeuchi and co-workers prepared MIP spheres for an atrazine template in a Y-junction microfluidics device with a length, width, and depth of 2 mm, 153.1 μm, and 80 μm, respectively [55]. This O/W system used 1.0 wt % poly (vinyl alcohol) (PVA) solution as the continuous phase. Atrazine, methacrylic acid (MAA), ethylene glycol dimetachrylate (EGDMA), and 2,2-azobis(2,4-dimethylvaleronitrile) were dissolved in mesitylene and utilized as the dispersed phase. The flow rates were fixed at 8 μL/min for the dispersed phase and 50 μL/min for the continuous phase using syringe pumps. The collected droplets were polymerized with UV light, and the diameter of the resultant polymer was approximately 50 μm (as discerned via SEM images). They used atrazine, an herbicide, as the target molecule. Since it is considered an endocrine disrupter, the removal of atrazine from the environment is of interest in environmental studies. Chromatographic experiments presented an evaluation of the selectivity of the atrazine-imprinted microspheres. Atrazine and the three other competitive molecules, namely, propazine, propyzamide, and thiuram, were analyzed in an HPLC system. Retention factors were calculated, for which it was revealed that the level of atrazine retained was higher than those of the other compounds. The results showed that the obtained particles from this novel method specifically rebound the target molecule and proved its potential to be used in various applications (Figure 6). The method demonstrates that polymeric microparticles can be used for the removal or separation of chemical or biological compounds of interest.

**Figure 7:**
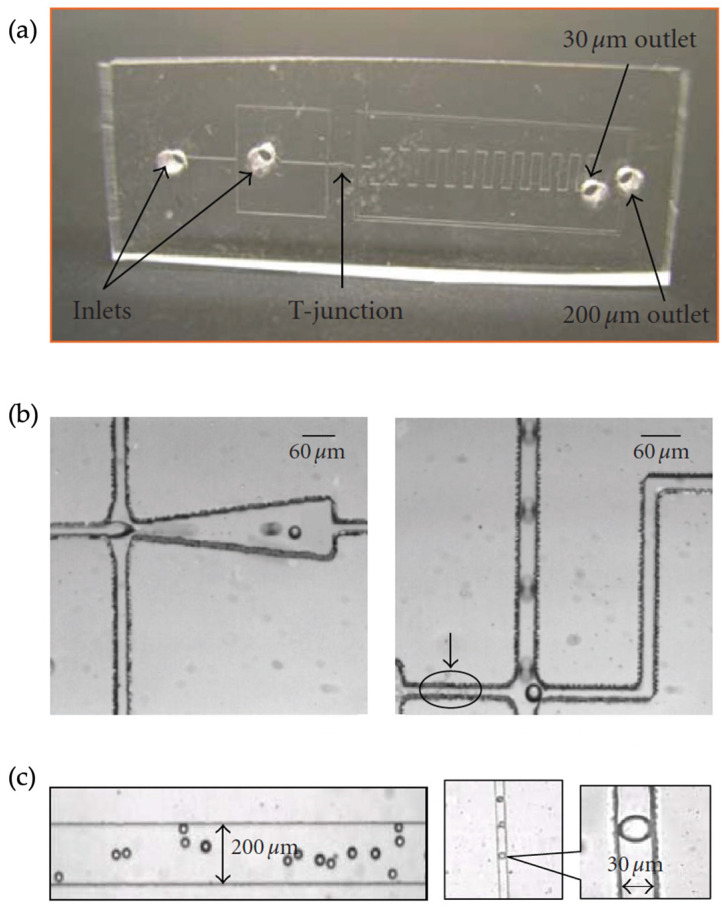
(**a**) Image of the PDMS microfluidic droplet generator, (**b**) video stills of droplet generation, and (**c**) uniform MIP droplets photocured in the channel. Reproduced with permission from [56].

Subsequently, a PDMS T-junction channel design was introduced in the literature to synthesize MIPs for a 9-ethyl adenine template [56]. The microfluidic channel has two inlets with dimensions of 30 μm in width and two outlets with dimensions of 30 μm and 200 μm. To create O/W droplets, the template molecule MAA, acting as the functional monomer; EGDMA, constituting the crosslinker; and 2-2-dimethoxy-2-phenyl-acetophenone, acting as the initiator, were dissolved in toluene. The flow rates were fixed at 1.0 μL/min for the dispersed phase and 2.5 μL/min for the continuous phase using syringe pumps. The dynamic motions of the resultant droplets were captured to demonstrate the formation of uniform MIP droplets almost 30 μm in diameter (Figure 7). Consequently, photopolymerization was completed before the droplets left the channel. They observed a significant difference in the refractive index between the cured and non-cured droplets. This study demonstrated the MIPs’ droplet formation in detail and their photocuring in the same microfluidic channel. There was no additional information about the resultant MIPs’ performance besides the use of microparticles as a high-affinity sensor for the target molecule.

**Figure 8:**
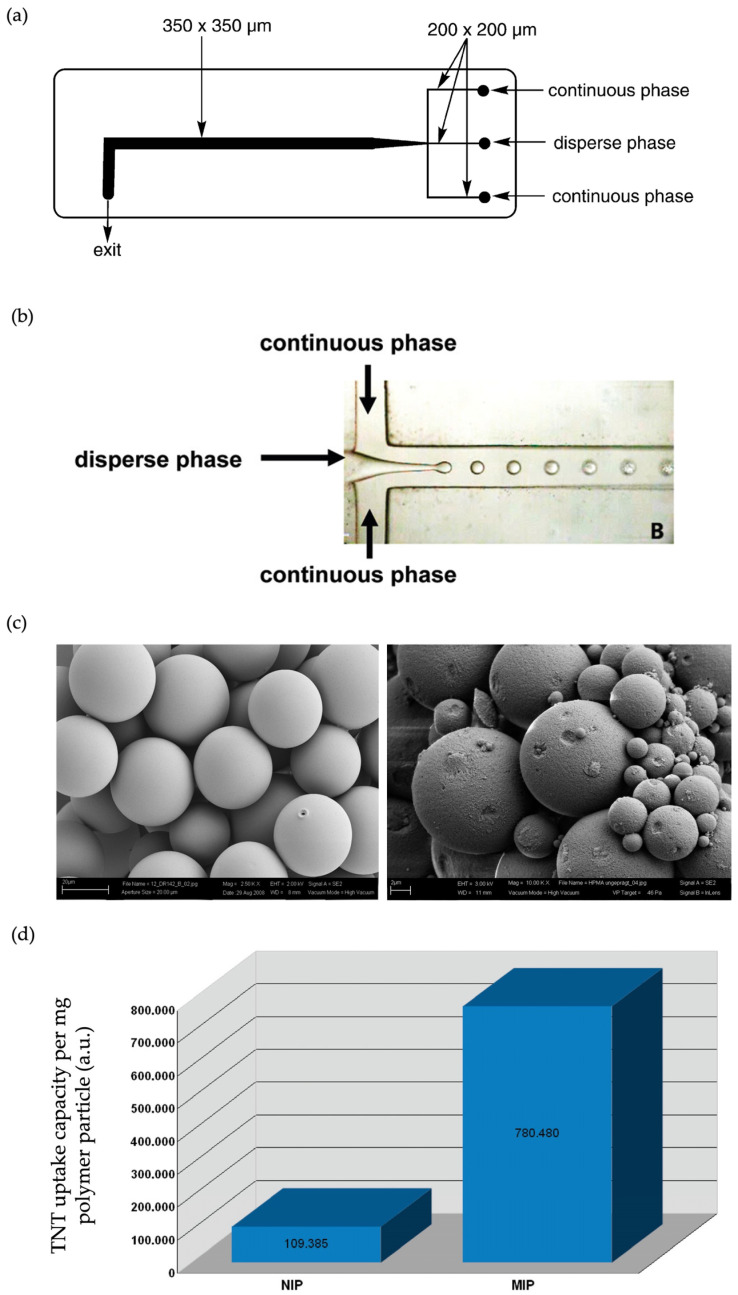
(**a**) Schematic demonstration of flow-focusing droplet generator; (**b**) a microscopic image of droplet generator; (**c**) SEM images of the MIP particles; (**d**) comparison of target molecule uptake by MIP and NIP. Reproduced with permission from [57].

A more comprehensive study was published by Roeseling et al. in 2009, wherein the authors utilized microparticles for explosive detection [57]. They successfully generated spherical droplets using two different types of channel design, namely, T-junction and flow-focusing, both of which were made of glass (Figure 8a,b). The particles were obtained using an O/W system with a 4% PVA containing water as the continuous phase. For the dispersed phase, a trinitrotoluene (TNT) template was dissolved in chloroform in the presence of a functional monomer (MAA), a crosslinker (EGDMA), and an initiator (Irgacure 819). A T-junction device was employed to obtain MIP and non-imprinted polymeric (NIP) microparticles. Batches of microparticles were prepared by fixing the flow rate of the continuous phase (Qc) at 0.4 mL/min and changing the flow rate of the dispersed phase (Qd) from 0.001 to 0.008 mL/min. The size of the resultant polymeric particles depended upon the flow rate ratio (Qc/Qd) and was in the range of 10 to 45 μm. The flow-focusing design was used to investigate higher flow rates for particle synthesis to increase the turnover of the droplet generator. The Qc/Qd was maintained identically to the experiments carried out in the T-junction device, but the flow rates for both the continuous and dispersed phases were increased. The authors observed that it is possible to tune the characteristics of the beads during the production step. For both setups, the obtained droplets were photocured using a UV source. The authors also explored the addition of PEG 32 kDa as a co-porogen to the dispersed phase. This resulted in increased porosity and an enlarged inner surface of the MIP particles as characterized by SEM (Figure 8c). The performance of the synthesized MIP beads as an explosive sensor was tested by comparing the TNT uptake of the NIP (Figure 8d). Overall, the authors successfully demonstrated that MIP-based technologies are beneficial for the creation of complex explosive-monitoring systems and can likely be extended to sense other target molecules.

**Figure 9:**
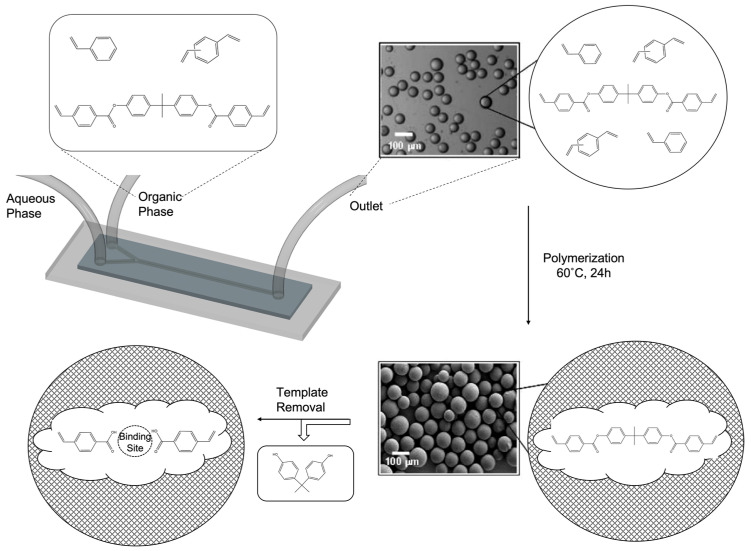
Schematic representation of Y-junction droplet generator used for BPA imprinted particles. Reproduced with permission from [58].

Another O/W attempt to create spherical MIP particles was reported by Takano et al. [58]. A Y-junction microfluidic device was prepared for the affinity-based solid phase extraction of Bisphenol A (BPA), which acts like an endocrine disruptor by interacting with estrogen receptors. A commercially available Y-junction microfluidic device with a width of 630 μm and a depth of 330 μm was operated by syringe pumps to obtain spherical BPA-MIP beads. As generally used in O/W setups, 5.0 wt % poly (vinyl alcohol) was used for the continuous phase and pumped at a varied range of 300 to 2200 μL/min. A Dichloromethane solution containing styrene, divinylbenzene, and 2,2′-azobis(2,4-dimethylvaleronitrile) was pumped through the channel as the dispersed phase with a fixed flow rate of 10 μL/min. The size of the particles was controlled by changing the flow rate of the continuous phase. The dimensions of the obtained particles were between 90–250 μm. For the solid phase extraction trails, 90 μm MIP beads were obtained by fixing the continuous phase’s flow rate at 2200 μL/min. The binding activity of the MIPs towards BPA and the competitive molecules, Bisphenol B and hexanol, were compared. It was found that BPA was more strongly bound to the MIP than the other molecules, thereby confirming that spherical BPA-MIP beads could be used in affinity-based SPE (Figure 9). The authors demonstrated how the BPA adsorption performance in solid phase extraction is changed by switching the eluent from polar to less-polar solvent systems. The results showed that BPA was not retained, and that elution was performed with 98% recovery. These results highlight that MIP particles are a good candidate for solid phase extraction applications.

**Figure 10:**
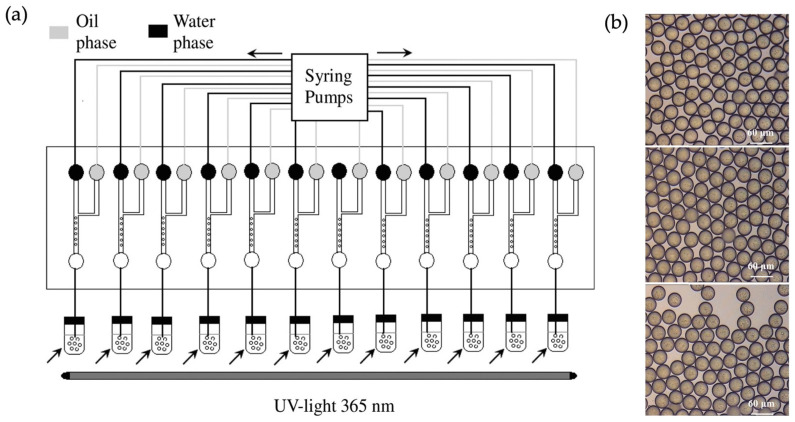
(**a**) Schematic illustration of 12 pairs of Y-shaped microfluidic channels; (**b**) optical microscopic images of droplets for different functional monomers (from top to bottom: MAA; 4-VP; AA). Reproduced with permission from [59].

Subsequently, a more effective production method for MIP particles was introduced in the literature via combinatorial synthesis and screening for simultaneous manufacturing [59]. The presented microfluidic design contains twelve pairs of Y-2-shaped microchannels used simultaneously to screen 12 kinds of imprinting conditions (as seen in Figure 10a). This technique merged molecular imprinting and the combinatorial chemical approach with a microfluidic design, enabling rapid screening, fast optimization, and high throughput. Chloramphenicol (CAP)-imprinted beads were prepared in an O/W system for the design trial. Water with 1.5% polyvinyl alcohol was used as the continuous phase, and an ethyl-acetate–chloroform (4:1, *v*/*v*) solvent system was utilized as the dispersed phase. The authors also optimized the molar ratio of the template molecule and the functional monomer and screened the final performances of three different functional monomers: methacrylic acid (MAA), 4-vinyl pyridine, and acrylamide (Figure 10b). Monodisperse polymer particles with an average size of 29 μm were obtained for all three functional monomers (as characterized by SEM). However, this study’s results demonstrated that MAA had the best performance for imprinting CAP with a molar ratio of 1:5 (CAP to MAA). Additionally, compared to a single-channel design, a larger amount of MIP beads was produced with twelve pairs of channels in a shorter time frame. The adsorption profiles of the three antibiotics, CAP, florfenicol (FF), and tetracycline (TET), were investigated to further evaluate the selectivity performance of the MIP beads. The imprinted beads had higher levels of CAP adsorption compared to the other antibiotics. This study indicates how microfluidics systems can be used for scaled-up applications combined with combinatorial protocols. It is a possible to design more than 12-pair channels, so this work demonstrates that the approach can be useful for high-throughput optimization and mass production in a commercial setting.

In 2014, Krupadam et al. introduced a microfluidic reactor setup for the fabrication of benzo[*a*]pyrene (BAP)-imprinted polymeric particles [60]. They utilized an O/W system with a 4% PVA solution in water as a continuous phase. As the dispersed phase, they used acetonitrile as the solvent, methacrylic acid as the functional monomer, and ethylene glycol dimetachrylate as the crosslinker. The droplets were generated in the microfluidics segmented flow process. Then, a UV source cured the formed acrylate droplets. During the droplets’ formation, the authors tuned the flow rates to obtain imprinted particles with different diameters. They kept the continuous phase at a certain flow rate and changed the dispersed phase’s flow rate. Consequently, they obtained MIP microspheres in a range between 60 to 500 μm. They also demonstrated the maximum turnover amount of the polymer microspheres per hour, which was 0.5 g. The obtained MIP particles demonstrated better binding capacity and selectivity toward the target analyte due to the well-defined binding sites. They aimed to achieve the selective extraction of polycyclic aromatic hydrocarbons from complex environmental samples and demonstrated the higher absorption capacity toward the target molecule over competitive compounds. There is great concern regarding industrial wastewater since it induces carcinogenicity and estrogenic activity, which lead to the development of cancer in humans. Therefore, the removal of pollutants from environmental samples is of paramount importance. Due to their stability in harsh environments, MIP-based particles are promising candidates for such environmental applications.

**Figure 11:**
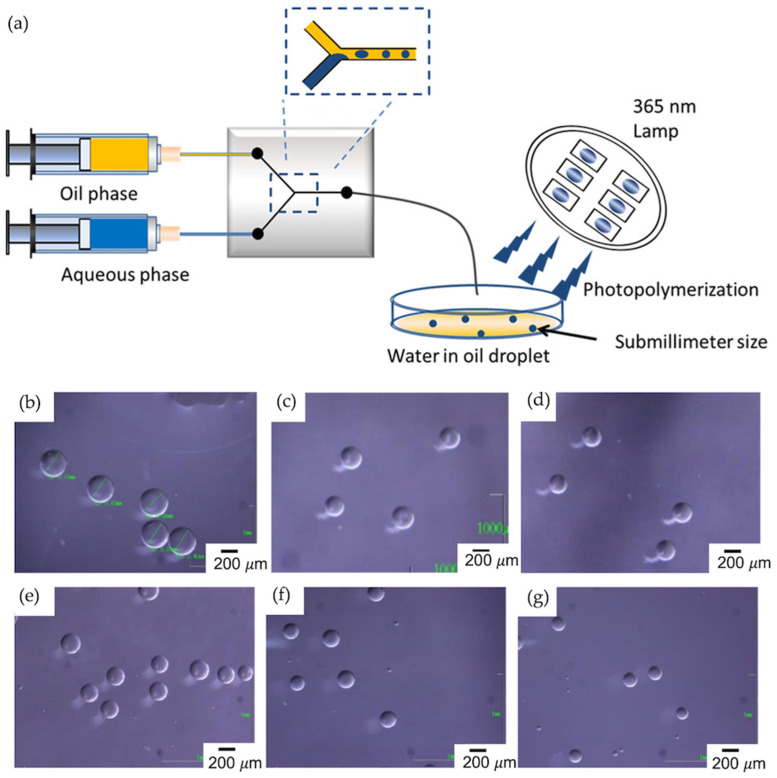
(**a**) General demonstration of Y-shaped microfluidic channel; (**b**–**g**) microscopic images of W/O droplets with different oil phase flow rates. Reproduced with permission from [61].

Most previously reported studies demonstrated droplet formation in an O/W system, for which the resultant beads were highly hydrophobic. Hydrophilic submillimeter-sized polymer particles are required to suppress nonspecific rebinding in applications. However, few attempts at and reports on particle generation through W/O-type droplets exist. Takeuchi and co-workers used W/O droplets containing a water-soluble monomer to generate MIP beads in a microfluidic channel for a specific protein target, Human Serum Albumin (HSA); this was the first attempt at the development of monodispersed submillimeter-sized MIP microgels synthesized in W/O droplets prepared using a microchannel [61]. A commercially available Y-shaped microfluidic channel that was 500 μm wide and 100 μm depth was utilized to produce beads (Figure 11a). For the continuous phase, mineral oil was used, and a suitable surfactant and its ideal concentration were investigated. The ideal surfactant was chosen by comparing Span 20, 40, and 85, among which it was indicated that Span 85 effectively formed stable droplets. The dispersed phase contained HSA as the template molecule, pyrrolidyl acrylate (PyA) as the functional monomer, acrylamide (AAm) and HEMA as comonomers, mBAA as the crosslinker, and Irgacure 2959 as the water-soluble initiator. The flow rates were fixed at 150 μL/min for the oil phase and 3 μL/min for the aqueous phase using syringe pumps. Before determining the ideal particle size, the authors optimized it by varying the flow rate of the dispersed phase, as seen in the microscopy images in Figure 11b–g. The collected droplets were photopolymerized at 365 nm overnight. Monodispersed particles with a size of 100 μm–1 mm with high hydrophilicity exhibited much higher affinity toward the target molecule compared to the non-imprinted submillimeter-sized particles. In addition, the selective binding profile of HSA-imprinted particles was investigated in the presence of the following reference proteins: BSA, chymotrypsin, and cytochrome c. The obtained submillimeter-sized particles with hydrophilicity are desirable for the development of proteomics studies since they can be used in affinity-column-packing studies to remove unwanted proteins before mass spectroscopy.

**Figure 12:**
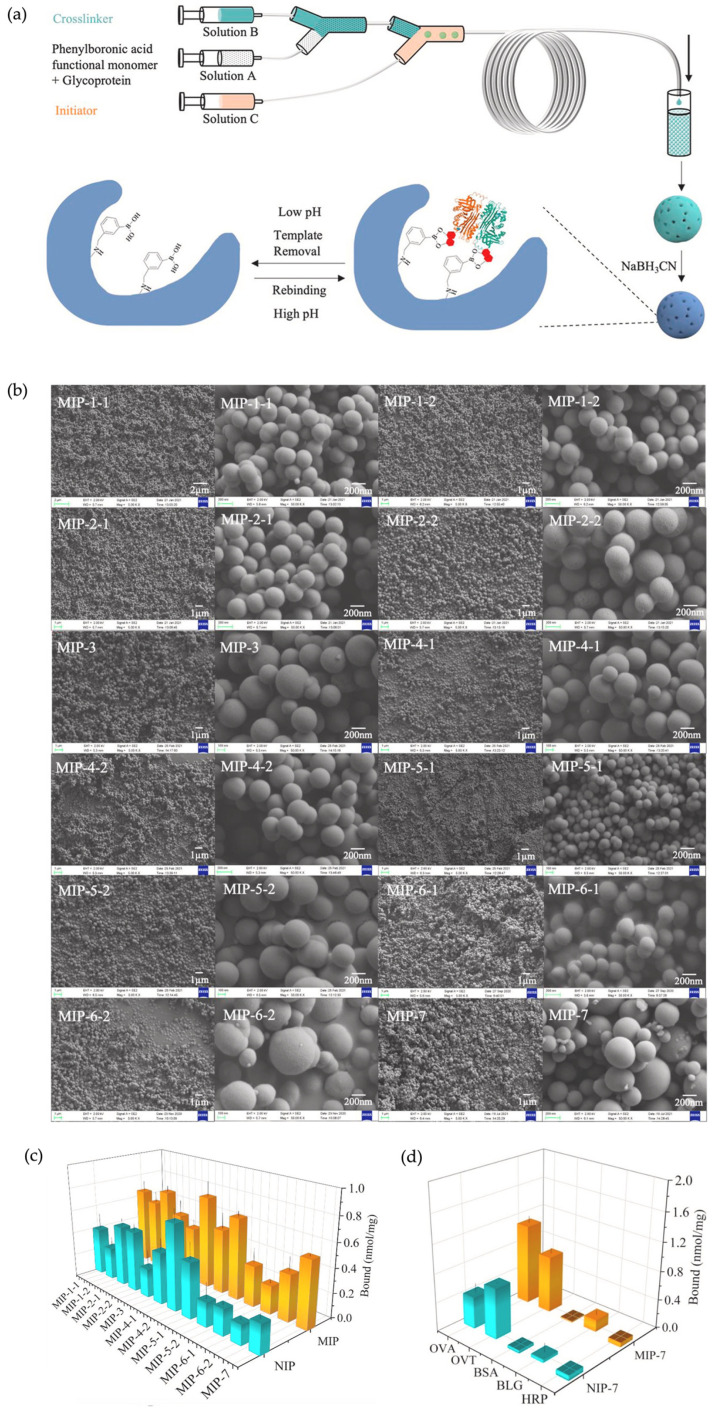
(**a**) Schematic illustration of microfluidics setup for molecularly imprinted glycoprotein droplets; (**b**) SEM images of the MIP nanospheres produced by the microfluidics setup via varying the fabrication conditions; (**c**) comparison chart of the MIPs regarding OVA uptake; (**d**) selectivity chart of the MIP nanospheres in the presence of competitive molecules. Reproduced with permission from [62].

Most recently, another W/O approach was presented by Jin et al. for the preparation of glycoprotein-imprinted nanospheres [62]. The authors utilized commercially available PTFE capillary columns as a microfluidics reactor setup. They investigated how nanospheres’ morphology, size, and selectivity are affected by adjusting the template, functional monomer, initiator concentrations, PTFE capillary column inner diameters, and flow rates. To prepare MIPs, three solutions (Solution A, B, and C) were used (Figure 12). While Solution A contained template molecule Ovalbumin (OVA) and phenylboronic acid as the functional monomer, Solution B was composed of tetraethyl orthosilicate (TEOS), which was employed as the crosslinker. A basic initiator of aqueous ammonia solution (NH_3_⋅H_2_O) was used as Solution C. The mixing of Solutions A and B with Solution C at the intersection of the capillary microreactor setup resulted in the formation of nanospheres. The obtained nanospheres were characterized by SEM and dynamic lighting scattering (DLS) and were found to be in the size range of 193–653 nm. A library of nanoparticles was obtained by adjusting the synthetic conditions to investigate how each condition impacts the morphology and size of the obtained nanospheres. Imprinted nanoparticles were produced in less than 2 h under the optimized conditions. They demonstrated good selectivity and specificity even in the presence of competing molecules, including horse-radish peroxidase (HRP), β-lactoglobulin (BLG), and bovine serum albumin (BSA). The extraction of ovalbumin was achieved with good selectivity and specificity. It was also demonstrated that the same MIP particles could be used for five cycles without losing their binding capacity. This study highlights that the implementation of a microfluidics-based design enabled the production of imprinted nanospheres with narrow dispersity and a high selectivity profile within a short timeframe.

## 4. Conclusions

While microfluidics devices continue to revolutionize the field of chemical and biological sensing and have become widely used as an efficient fabrication tool for obtaining microparticles, applications in the area of molecularly imprinted polymers remain limited. MIP-based technologies have great potential for use as commercial systems due to their long shelf-life and chemical stability [63,64], and their integration with microfluidics technology is bound to expand and streamline their applications. Additionally, scaling up the high throughput production rate of droplet-based microfluidics using different methods, such as parallelization or incorporating multiple microfluidic channels on a single chip, is possible and would be pragmatic for parallel analysis. Importantly, MIP-based microparticles can be produced on a large scale using a simple setup and thus can be easily scaled up for widespread application. Most microfluidics-based imprinted particle fabrications are undertaken at room temperature under mild conditions. This is an attractive asset since the conditions are conducive to an efficient templating process when compared to the moderately high temperatures used in many of the traditional and conventional approaches for particle formation during polymerization. Another attractive attribute of microfluidics-based fabrication is that rapid optimization can be achieved using low amounts of materials. Thus, a combinatorial approach to the preparation of MIP micro/nanoparticles for solid phase extraction columns, separation studies, and biological/chemical sensors can be utilized to find the best formulation parameters to obtain the most suitable candidate. In addition, advances in polymerization techniques and microfluidic device design will also yield improvements in the tailoring of the size and dispersity of such particles over a large scale. We hope that the handful of examples discussed in this review highlight the untapped potential of the microfluidics-based approach for the acquisition of MIP-based microparticles, which can serve as the best candidate materials for applications in various areas of biomedical sciences. 

## Figures and Tables

**Table 1:** Comparison of different preparation methods of MIPs.

Methods Used for MIP Synthesis	Advantages	Disadvantages	Refs.
Bulk Polymerization	-Simple preparation -Low cost -No extra solvents are required	-A grinding process is required -Destroys binding sites -Particles are irregularly shaped -Not suitable for imprinting large molecules	[16,17,18]
Precipitation Polymerization	-MIP beads with regular shape -Higher yield -Polymeric chains grow individually -Easy procedure	-Excessive amount of solvent required	[19,20,21,22]
Suspension Polymerization	-MIP beads with regular shape -Larger particle size suitable for SPE	-Surfactant contamination -Reaction requires both organic and aqueous phases	[23,24,25,26]
Multi-Step Swelling	-Monodispersed beads in size and shape -Well suited for chromatographic applications	-Complex polymerization procedures and reaction conditions are not desirable -Competing solvent effect	[27,28,29]
Core–Shell Grafting	-Higher adsorption capacity -Uniform distribution of binding sites -Faster mass transfer	-Complex synthesis procedure -Aggregation	[30,31,32,33,34]

**Table 2:** Summary of studies using droplet-based microfluidics to prepare MIP particles.

Template	Channel Geometry	O/W or W/O	Application	Particle Size	Refs.
R,S-propranolol	Spiral shape reactor	W/O	Binding Assay	25 μm	[54]
Atrazine	Y-junction	O/W	Separation	50 μm	[55]
9-ethyl adenine	Y-junction	O/W	Sensor	30 μm	[56]
Trinitrotoluene	T-junction	O/W	Explosive Detection	10–45 μm	[57]
Bisphenol-A	Y and T-junction	O/W	Solid-Phase Extraction	90–250 μm	[58]
Chloramphenicol	Y-junction (12-pairs)	O/W	Antibiotic Detection	29 μm	[59]
Benzo[*a*]pyrene	Microfluidic reactor setup	O/W	Extraction	50–500 μm	[60]
Human serum albumin	Y-junction	W/O	Protein Removal	100 μm–1 mm	[61]
Ovalbumin	Y-junction	W/O	Extraction	193–605 nm	[62]

## Data Availability

Not applicable.
